# Grandmotherhood across the demographic transition

**DOI:** 10.1371/journal.pone.0200963

**Published:** 2018-07-23

**Authors:** Simon N. Chapman, Jenni E. Pettay, Mirkka Lahdenperä, Virpi Lummaa

**Affiliations:** Department of Biology, University of Turku, Turku, Finland; TNO, NETHERLANDS

## Abstract

Grandmothers provide key care to their grandchildren in both contemporary and historic human populations. The length of the grandmother-grandchild relationship provides a basis for such interactions, but its variation and determinants have rarely been studied in different contexts, despite changes in age-specific mortality and fertility rates likely having affected grandmotherhood patterns across the demographic transition. Understanding how often and long grandmothers have been available for their grandchildren in different conditions may help explain the large differences between grandmaternal effects found in different societies, and is vital for developing theories concerning the evolution of menopause, post-reproductive longevity, and family living. Using an extensive genealogical dataset from Finland spanning the demographic transition, we quantify the length of grandmotherhood and its determinants from 1790–1959. We found that shared time between grandmothers and grandchildren was consistently low before the demographic transition, only increasing greatly during the 20^th^ century. Whilst reduced childhood mortality and increasing adult longevity had a role in this change, grandmaternal age at birth remained consistent across the study period. Our findings further understanding of the temporal context of grandmother-grandchild relationships, and emphasise the need to consider the demography of grandmotherhood in a number of disciplines, including biology (e.g. evolution of the family), sociology (e.g. changing family structures), population health (e.g. changing age structures), and economics (e.g. workforce retention).

## Introduction

In cooperatively-breeding species across a range of taxa, offspring are cared for not only by the parents, but also by other available group members (i.e. alloparents). Who helps in such cooperative societies is determined by a range of factors affecting helper willingness to help, including relatedness [1] and ecological conditions [2], as well as those that may affect the availability of different alloparents to provide care, such as sex differences in dispersal or lifespan. In human societies, alloparents may include unrelated individuals, such as day-carers, but more traditionally are female relatives, such as grandmothers [3], aunts [4–6], and siblings [4,5,7–9]. Which relatives help and how much time they invest in child care varies considerably across studied populations [3], but few studies have addressed the causes and underlying factors of such variation.

Grandmothers have historically been and continue to be important alloparents in human families, with benefits of help including grandchildren’s greater probability of surviving childhood [3,10–13], improved mental health [14,15], possibly enhanced cognitive development [14], and better nutrition [16,17]. However, large differences across the studies on grandmother effects have been noted: grandmother presence may sometimes also be associated with negative outcomes for grandchildren, such as reduced survival [18,19], which can occur when grandmothers are themselves still reproductive [20]. Conversely, there are also both beneficial and detrimental effects of grandparenting to grandmothers themselves. Grandmother physical and mental health, for example, can be affected by the level of care they provide to grandchildren [21,22]; grandmother happiness in Finland positively correlates with increasing contact [23], whilst in the US, those co-residing with a grandchild are more likely to suffer from depressive symptoms [24]. Additionally, caregiving grandparents in contemporary Germany had a decreased mortality risk compared to non-caregiving grandparents and to non-grandparents [25], albeit grandmothers were not distinguished from grandfathers in this study. Whether such health effects are a universal condition of grandmothering in humans and are common across different conditions is unknown at present, as most studies on the health impacts of grandmothering are confined to affluent industrialised countries.

Critically, these effects of grandmothering on grandchildren and on the grandmothers themselves are constrained by the time that both are simultaneously alive, and quantifying this shared time is therefore of great importance. For example, between-population differences in the length of shared time may underlie some of the observed heterogeneity in grandmother-mediated effects between populations. Similarly, differential effects of maternal and paternal grandmothers on grandchild health or survival seen in many populations [3] may be due in part to lineage differences in how long maternal vs paternal grandmothers are present for their grandchildren. Finally, the beneficial and adverse effects from intergenerational relationships between grandmothers and subsequent generations have been linked to the evolution of menopause and prolonged post-reproductive life in humans [10,12,20,26], but developing such theories further and measuring the strength of selection on these traits strongly depends on quantifying the availability of grandmothers to grandchildren in different settings. Even large grandmother benefits to grandchild outcomes would be of little evolutionary value if only a few grandmothers in our evolutionary past lived beyond the critical first years of their grandchildren’s lives and if few grandchildren had any grandmother available to them in the early years (as is the case in some other species with measurable grandmother benefits but only a small fraction of grandoffspring benefitting from them, such as Japanese macaques *Macaca fuscata* [27]). To address this shortcoming, understanding how the shared time between grandmothers and their grandchildren in contemporary societies reflects past conditions, or varies between populations, would be of considerable value.

Many factors may contribute to geographic and temporal variation in grandmotherhood length. Early death of either the grandmother or the grandchild sets an absolute limit to shared time, but geographical variation and differing time-trends in old-age longevity and infant mortality are not the only reasons for heterogeneity in grandmotherhood length. Changes in the timing of reproduction and family size can also limit the theoretical window for grandmother help [28–30] by affecting e.g. grandmother age at grandchild birth and grandchild birth order: later-born grandchildren will have less time with their grandmothers. We know little of the length of grandmotherhood in pre-industrial societies, other than it was likely to have been shorter than today, because few studies have investigated past trends [29,31,32], especially before the 20^th^ century. Understanding the temporal changes in the length of grandmotherhood and availability of grandmothers is needed for deeper understanding of current intergenerational relations and of forces that may have shaped these relationships. Such information could also have importance in different fields, including biology, economics, and population health [28]. For example, quantifying how the grandmother-grandchild relationship changed through time would be useful for evolutionary studies into family formation and the maintenance of cooperation in multi-generational societies, whilst mental and physical health of the elderly is becoming increasingly important for medical fields as demographic structures in many populations are skewing towards older ages. Sociological research into families could also benefit from research into shared times between grandmothers and grandchildren as the importance of nuclear families decreases and the importance of multigenerational relations increases [33], especially for grandchild outcomes; grandmothers can provide stability for their grandchildren during psychologically difficult family events e.g. parental divorce [33].

Using register data from the Lutheran Church in Finland, we explore the changing demography of the grandmother-grandchild relationship over more than a century and a half, for birth cohorts of grandchildren born between 1790 and 1959 (170 years; 66160 grandchildren, 7349 grandmothers). During this time period, childhood mortality and fertility rates were initially high [34,35], then declined with the progress of industrialisation and the demographic transition to current low mortality and fertility rates, which began from around the 1870s in Finland [34,36]. This dataset and study period therefore offers an exceptional opportunity to examine the grandmother-grandchild relationship before and during a time when major shifts were occurring in population level traits and in society at large. We limited our study to grandmothers only, as the shared time of grandfathers was negligible in pre-modern Finland [32], and because grandfathers in this population were of little importance to grandchild outcomes [37]. We also studied both maternal and paternal grandmothers, as many populations with known grandmother effects show a difference in these effects [3]. Furthermore, we investigated aspects of the grandmother-grandchild relationship from both the grandmother and grandchild perspective, as the nature of the relationship is markedly different for each: grandmothers may have multiple grandchildren for whom they may split investment, whilst grandchildren have two grandmothers, and the death of one does not prevent continued helping investments and associated benefits from the other.

First, we quantify the proportion of grandchildren with a grandmother alive at birth for both lineages, and how this changed across time, in order to provide a basis for how many grandchildren had an opportunity to interact with their grandmother. Then, we investigate changes in the shared time between grandmothers and grandchildren, to quantify the absolute time limit for potential to interact. These two measures are the most important for intergenerational interactions, but it is also important to investigate factors that underlie changes in these measures. To test possible drivers for observed shifts in shared time, we then quantify changes in both the percentage of grandchildren outliving grandmothers, as a proxy for changes in mortality patterns, and grandmother age at the birth of a grandchild, to ascertain whether fertility decisions may have affected the shared time. Following this, we quantify changes in the average number of grandchildren born to a grandmother before her death and in total, as the number of grandchildren a grandmother had during her life could affect the costs and benefits of grandmothering to herself (e.g. more exhaustion) and also to her grandchildren (e.g. if the grandmother is alive for more grandchildren, she may lower investment in each grandchild, but more grandchildren will potentially receive care). Finally, we also quantify changing patterns in the proximity of living locations of grandchildren and grandmothers, as an indicator of the potential for grandmothers to directly invest in their grandchildren and of familial dispersal: parents may have stayed close to their mothers to facilitate grandmother help.

## Methods

### Study population

Our study dataset originates from parish registers from the Lutheran Church, and from published genealogies for individuals and their descendants, from eight Finnish parishes (Hiittinen, Rymättylä, Tyrvää, Ikaalinen, Pulkkila, Jaakkima, Rautu, and Kustavi). It must therefore be noted that the values we report may not be applicable to the whole of Finland, as mortality and fertility rates locally vary [38,39]. These records were maintained by local clergymen for the entire population of Finland from 1749 [40], and detail key demographic events including births, deaths, marriages, dispersal, and occupations. Divorce was forbidden in historical Finland and adultery punished [41], so extra-pair offspring and paternity uncertainty was likely to have been low. We are therefore able to construct pedigrees of up to 15 generations [42], and follow the life events of many individuals and their descendants.

Finland before the demographic transition (progression from high child mortality and high fertility to contemporary low birth and death rates) and industrialisation was predominantly agrarian, with the economy heavily based around agriculture and fishing. Farming techniques were relatively primitive though [43], and industrialisation came far later than the rest of Europe, only beginning in the latter part of the 1800s [36], and continuing into the 20^th^ century. Household sizes were often large, with many related individuals co-residing [44,45]; it was not uncommon to find multiple generations living together, which may have facilitated intergenerational interactions. Pre-industrial medical care was characterised not only by typically low standards of hygiene (due to a lack of understanding on how diseases were spread), but also by an exceptionally low ratio of academically-trained physicians to the population size [46]. Industrialisation brought about better medical care and higher standards of hygiene and coincided in Finland with the introduction of smallpox vaccinations, and this may in part have catalysed the demographic transition by reducing mortality rates. The demographic transition itself began in the 1870s [34].

We included in this study individuals born between 1790 and 1959 for whom birth information and information about at least one grandmother was known (specific information about sample selection can be found under each analysis sub-section). Individuals were separated into birth cohorts of ten years (i.e. 1790–1799 and so forth), allowing quantification of changes through time. There were four notable historical events covered in our study period that could have affected the length of the relationship between grandmothers and grandchildren in our study areas: Finland becoming part of the Russian Empire in a war of 1808–1809, a devastating famine from 1867–1868 [47], Finnish independence and civil war in 1917–18, and cessation of eastern regions of the country to the Soviet Union after two wars fought 1939–1944.

### Statistical analysis

All analysis was undertaken in R 3.3.1 [48]. Unless otherwise specified, all sample sizes are of grandchildren. Sample sizes differ between analyses due to the way censored individuals were differently handled in each analysis, which is explained in the relevant sections. Sample sizes also differ between maternal and paternal grandmothers within each analysis due to incompleteness in the registers; for example, occasionally the identity of only one grandmother is known, and therefore the counterpart could not be included in the other grandmother-grandchild dyad.

Time trends were statistically tested using Spearman’s rank correlation coefficient, between model-predicted values or percentages (depending on the analysis; see below), and birth cohort. Birth cohort was ranked from 1950s backwards (i.e. 1950–1959 is 1^st^, 1940–1949 2^nd^, and 1790–1799 would be 17^th^), and the variable of interest in each analysis (e.g. shared time) ranked with the highest value as 1^st^ and the lowest as 17^th^; a positive correlation indicates an increase through time. These tests were implemented with *cor*.*test*, which corrects for ties.

#### Percentage of grandchildren with a grandmother alive at birth

The percentages of grandchildren with a grandmother alive at birth can indicate the relative population-wide potential for grandmother-grandchild interactions. As such, we calculated these figures for each birth cohort for the maternal grandmother-grandchild dyad (n = 37788) and the paternal grandmother-grandchild dyad (n = 35954). We excluded only those whose maternal or paternal grandmother had no date of death and had last been recorded in the registers before the birth of the grandchild in question (i.e. the grandmother was censored before the birth of the grandchild).

#### Shared time between grandmothers and grandchildren

Length of shared time (from grandchild birth to death of either the grandchild or the grandmother) was quantified to investigate how the limits of the (potential) grandmother-grandchild relationship changed across the demographic transition. Shared time was calculated across life for all individuals (maternal grandmother analysis: n = 38394 individuals, 5260 maternal grandmothers; paternal grandmother analysis: n = 36268 individuals, 4915 paternal grandmothers) and not limited to only the childhood of individuals. If the first person in the dyad (grandmother or grandchild) to disappear had no date of death, the length of time in years was censored at the year of disappearance. Grandmothers who had died before birth of a grandchild were included in this model, with their shared time set as 0 years. Grandmothers who had disappeared before birth of a grandchild had their shared time set as 0 years as well, but were assigned a zero on the event status variable to indicate that ‘failure’ (i.e. death) was not observed. We note that some of the grandmothers who disappeared may have survived after the birth of the grandchild, which potentially biases our estimates downwards. Since the number of grandparents who disappeared before birth is less than 10%, any biases due to missing dates of death are likely to be small in comparison to our estimates of historical changes in shared time. We also assessed length of shared time for those with a grandmother alive at their birth (maternal grandmother analysis: n = 14829, 2819 maternal grandmothers; paternal grandmother analysis: n = 16550, 2904 paternal grandmothers). We then used this information in separate cox proportional hazards models, which can account for censoring, for maternal grandmothers and paternal grandmothers. These were implemented with functions from the R package *survival* [49], with a response variable of the length of shared time and an explanatory variable of birth cohort. From these, and using the *survfit* function from *survival*, we were able to obtain the average length of shared time for each cohort for the maternal and paternal grandmother-grandchild dyads.

#### Children outliving grandmothers

We calculated the percentage of grandchildren outliving their grandmothers (a proxy for childhood mortality and/or grandmother longevity), to quantify how mortality patterns changed over time, and in turn how this may have affected the shared time of grandmother-grandchild relationships. Pre-deceasing a grandmother is indicative of either childhood mortality, or grandmother longevity, depending on whether death occurs in childhood or adulthood. Due to some individuals lacking a death record, we excluded individuals when they a) died after a grandmother who did not have a recorded date of death (and was thus censored), b) did not have a recorded date of death themselves, and neither did the grandmother (both were censored), or c) had no recorded date of death but were last recorded in the church registers before the grandmother died. We then obtained percentages of grandchildren outliving their grandmothers for each cohort, separately for maternal grandmothers (total n = 31224) and paternal grandmothers (total n = 30625).

#### Grandmother age at birth

To investigate whether fertility decisions may have affected any changes in the length of shared time between grandmothers and grandchildren, we calculated the grandmother age at grandchild birth across time. Grandmother age at the birth of grandchildren is a fertility-related trait that can have a clear impact on the length of grandmothering time: the earlier births are, the more likely it is that grandmothers are a) alive, and b) alive for more of a child’s life. Therefore, we investigated how grandmother age at grandchild birth has changed: a) age at which a grandmother first becomes a grandmother (maternal grandmother n = 5384; paternal grandmother n = 5023), and b) average grandmother age for all births (maternal grandmother n = 39319; paternal grandmother n = 37209). These were then used as response variables in a series of cox models (see above), again with grandchild birth cohort as the explanatory variable, to investigate how grandmother age at grandchild birth may have changed over time.

#### Number of grandchildren

To investigate how the number of grandchildren a grandmother had might have changed, which could affect the level of investment in each grandchild, we selected all grandmothers in our sample (n = 7264), rather than all grandchildren (as in previous sections). Birth cohort was determined by the decade in which a grandmother’s first grandchild was born. We then implemented cox models, with the response variable as total number of grandchildren ever born to the grandmother in one model (irrespective of when she died), and number born during grandmother lifetime in the other. The explanatory factor was set as birth cohort (17 levels). We were able to extract the average total number of grandchildren and average number of grandchildren born during a grandmother’s life from these models.

#### Proximity to grandchildren

To provide further indication of how much living grandmothers might have been able to provide help through time, we calculated the percentages of grandchildren living close to or far from their maternal (n = 37862) or paternal grandmother (n = 36135) across each birth cohort. First, we quantified whether grandmothers and grandchildren lived in the same parish, or a neighbouring or different parish based on the birth parish of a grandchild and the living parish of their grandmother, irrespective of the survival status of the grandmother. A neighbouring parish was counted as any parish bordering the birth parish of the grandchild, and any other parish was considered ‘different’. If a grandmother had missing parish information for her adulthood, grandmother location was taken to be her birth parish, and this was used instead; a grandchild and grandmother born in the same parish indicates dispersal of the family was low. We included neighbouring parishes as a category to account for administrative change over time, as new parishes were established or borders changed. For example, the parish of Parkano got its charter in 1867 and was separated from the larger parish of Ikaalinen. We obtained percentages for each classification (same parish, neighbouring parish, different parish) in each birth cohort.

## Results

### Length of intergenerational relationships

#### Percentage of grandchildren with a grandmother alive at birth

**Fig 1:**
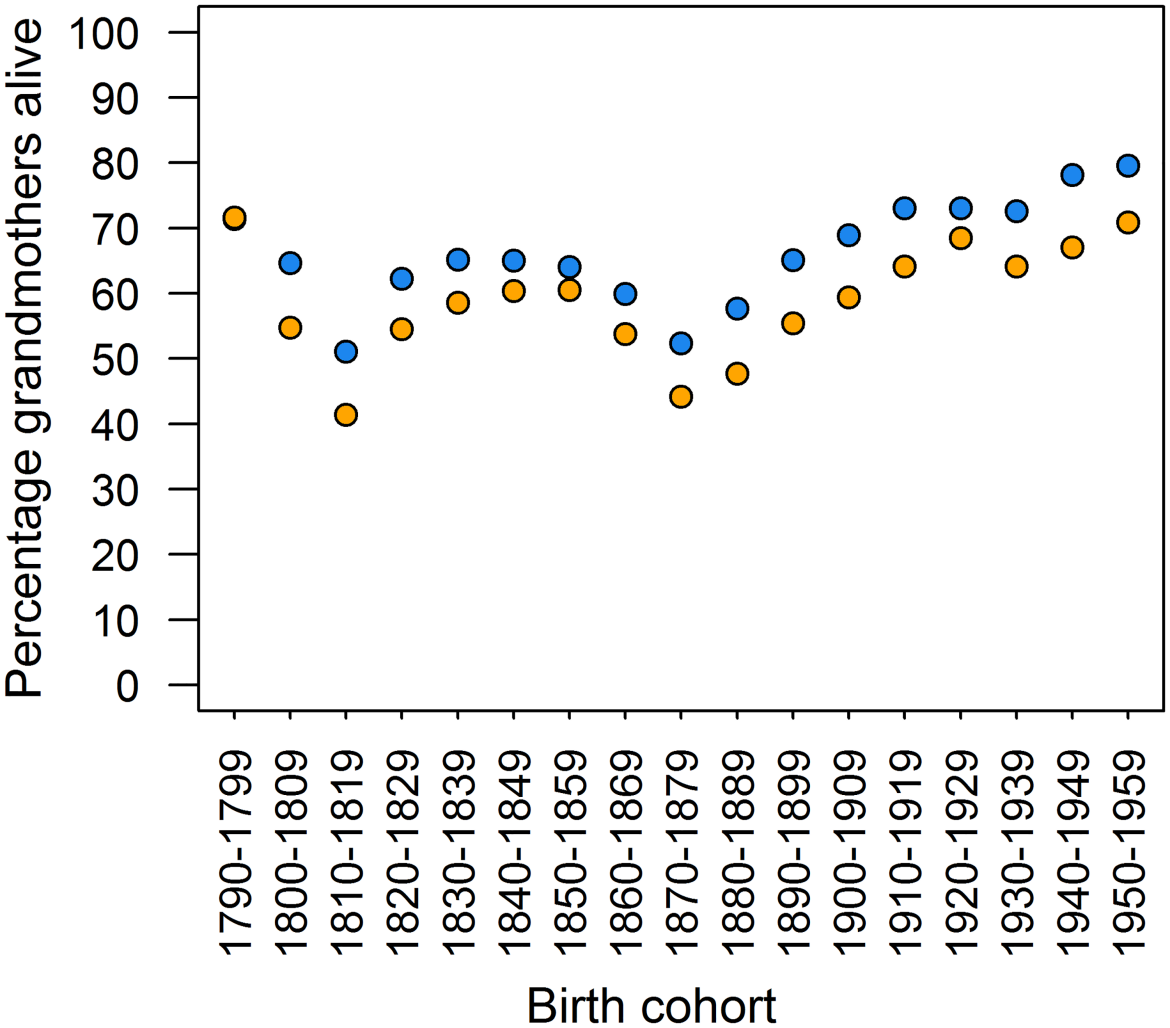
Percentage of individuals with a grandmother alive at birth. Blue represents maternal grandmothers, orange represents paternal grandmothers.

We first assessed the percentage of individuals with a grandmother alive at birth and through their childhood, and found evidence that more than a third of grandchildren across the entire study period in our sample were not able to interact at all with their grandmother: 36.2% of children were born without a living maternal grandmother, and 43.6% without a living paternal grandmother. The percentage of children born with a living maternal grandmother increased through time (Spearman’s rank r_s_ = 0.625, p = 0.009). For much of the 19^th^ century, greater than 65% of children had a living maternal grandmother at their birth (Fig 1), with lows of 51.4% in the 1810s and 52.4% in the 1870s—grandmothers from these cohorts may well have died as a consequence of the 1808–1809 war and the mid-1860s famine. The percentage then increased to a high of 79.6% for those born in the 1950s. For paternal grandmothers, the pattern was similar (r_s_ = 0.427, p = 0.089; Fig 1), albeit with a lower percentage alive at birth—greater than 54% for much of the 19^th^ century, with lows of 41.4% in the 1810s and 44.2% in the 1870s. From the 1900s, more children were born with a living paternal grandmother, rising to 70.9% in the 1950s.

#### Shared time between grandmothers and grandchildren

We also found evidence that the average potential grandmother investment period, measured by the shared time of grandchildren and their grandmothers (when both alive) was very low until the 20^th^ century, even for those grandchildren who were born when their grandmother was still alive.

**Fig 2:**
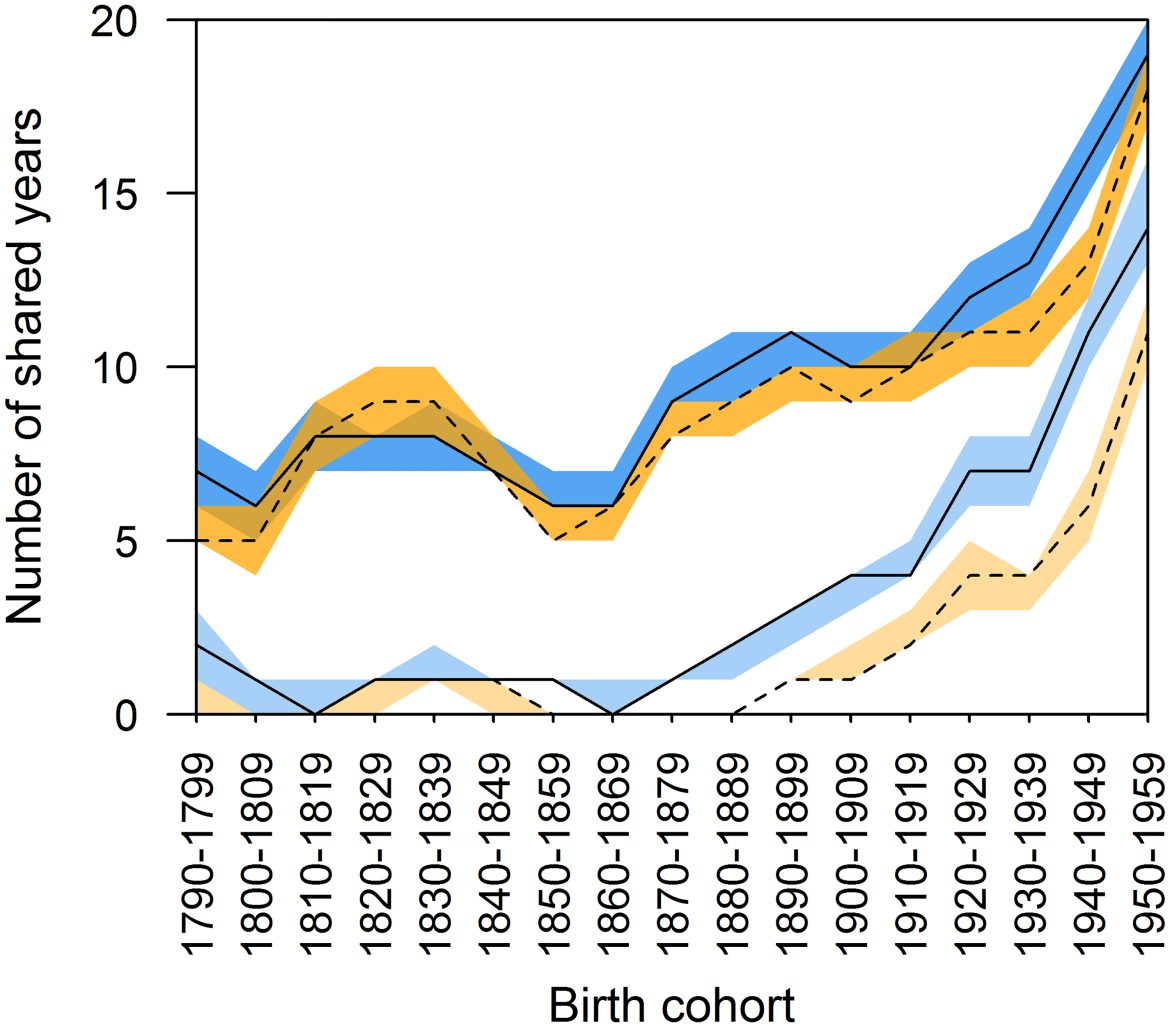
Shared time between grandmothers and grandchildren. Maternal grandmothers are shown with solid lines and blue 95% confidence intervals, paternal grandmothers with dashed lines and orange 95% confidence intervals. Lighter colours indicate the shared number of years for all individuals, darker for only those with a living grandmother at birth.

The average shared time of all grandmothers and grandchildren (including grandmothers who had died before the birth of a grandchild) was exceptionally low for both maternal and paternal grandmothers during the 19^th^ century (Fig 2). It never rose above 1 year for paternal grandmothers during this time and varied between 0 and 2 years for maternal grandmothers up to the 1870s, and then began to rise to an average of 4 years for cohorts born in the beginning of the 20^th^ century. This increase continued throughout the study period (r_s_ = 0.815, p<0.001), reaching a high of 14 years in the 1950s for maternal grandmothers. Similarly, paternal grandmother-grandchild shared time increased (r_s_ = 0.766, p<0.001), albeit starting from the 1880s, and reached a high of 11 years by the end of this period. Such low numbers were largely driven by a low percentage of grandmothers alive at grandchild birth (see Methods for possible influence of selection bias).

The number of years a grandmother and grandchild would share when the grandmother was alive at birth was higher than the average shared time for all individuals, but was still low compared to contemporary populations: between 5 and 10 years for nearly all the pre-20^th^ century birth cohorts. Shared time increased greatly (maternal grandmothers r_s_ = 0.859, p<0.001; paternal grandmothers r_s_ = 0.850, p<0.001), starting around the onset of the demographic transition in the 1870s for both maternal and paternal grandmothers, reaching highs of 19 and 18 years respectively for the 1950s birth cohort.

### Possible drivers of intergenerational relationship length

#### Children outliving grandmothers

Our analysis of the timing of grandchild and grandmother death patterns through time indicated that improving grandchild survival, rather than grandmother longevity, accounted for most of the observed increase in the period of potential grandmother investment.

**Fig 3:**
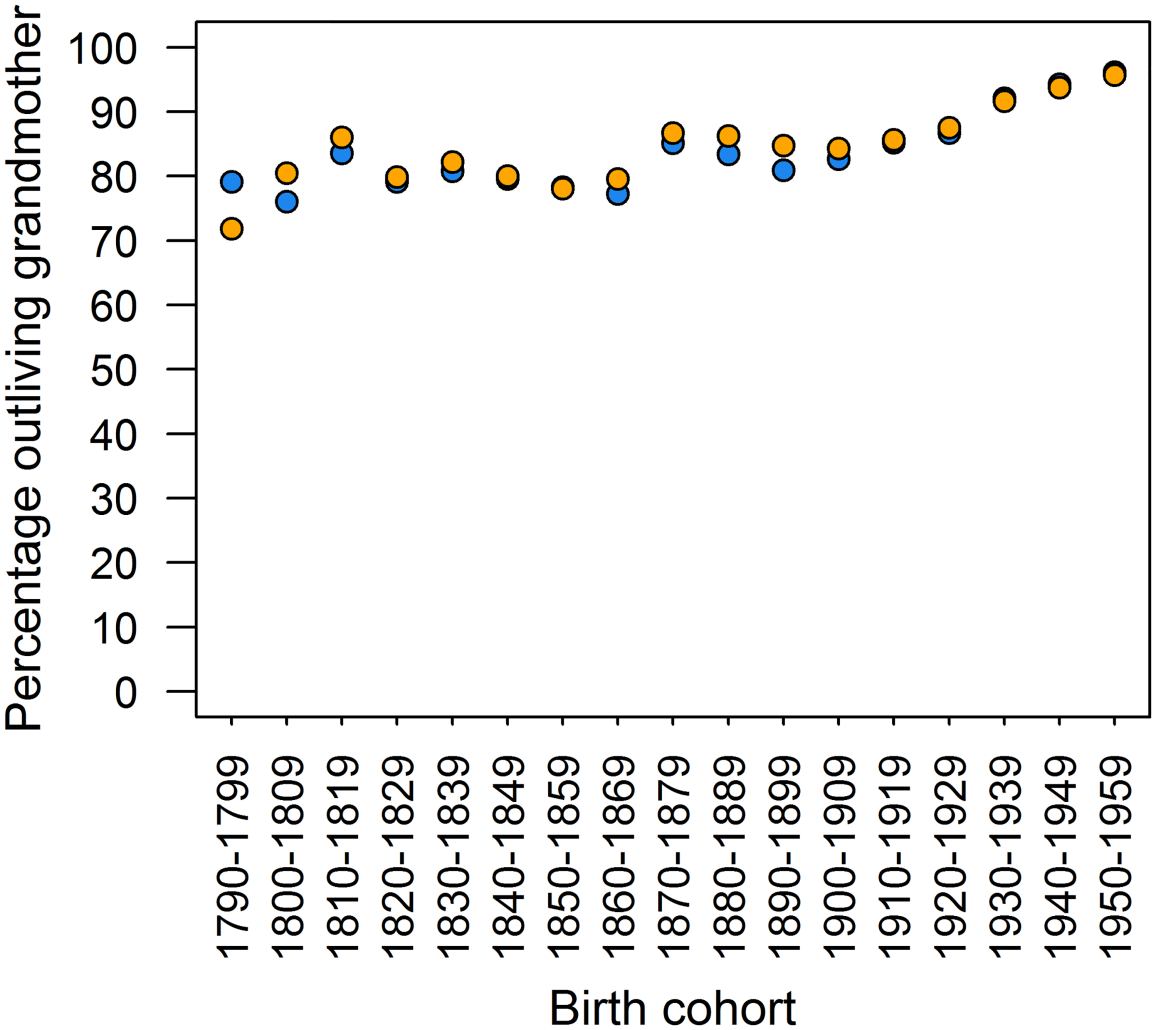
Grandchildren outliving grandmothers. Orange circles represent paternal grandmothers, and blue maternal grandmothers.

Over the entire period, the majority of grandchildren outlived their grandmother (Fig 3): more than 80% in every cohort from the start of the demographic transition (1870s) for both maternal and paternal grandmothers. There was little difference in the percentages between grandmother lineages in the 20^th^ century: both reached similar highs in the 1950s, with 96.2% outliving a maternal grandmother and 95.7% outliving a paternal grandmother. Again, there were increases overall from the 1790s to 1950s cohorts for maternal grandmothers (r_s_ = 0.806, p<0.001) and for paternal grandmothers (r_s_ = 0.775, p<0.001). Across the study period, 17.7% of grandchildren pre-deceased their maternal grandmother, and 15.9% their paternal grandmother. Of these deaths, 95.7% and 93.5% (for maternal and paternal grandchildren respectively) died in childhood, before 15 years, suggesting that changes in the percentages of children surviving longer than a grandmother is related more to decreasing childhood mortality than to increasing grandmother longevity.

#### Grandmother age at birth

**Fig 4:**
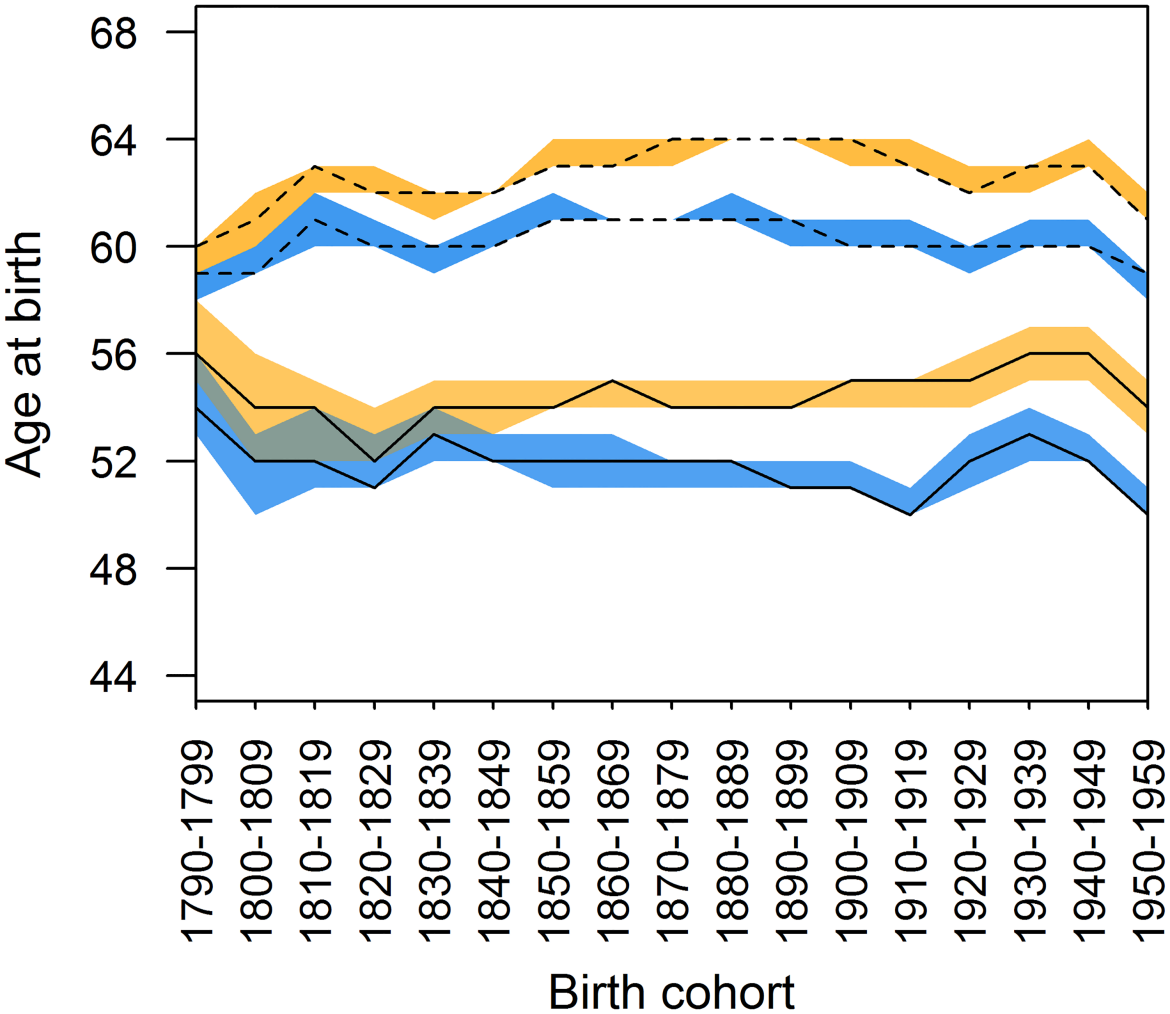
Average age at birth for grandmothers. Solid lines represent average age at first grandchild birth, and dashed for average age at the birth of any grandchild. Maternal grandmothers are represented by lines within blue 95% confidence intervals, and paternal grandmothers by lines within orange 95% confidence intervals.

We next investigated whether changing maternal birth scheduling patterns may have accounted for any of the changes we observed in the length of grandparenthood, but found little evidence for this. Grandmother age at the birth of grandchildren changed little over the 170 year period (Fig 4): the average grandparental age at first birth was fairly consistent across the full study period for both maternal and paternal grandmothers (daughter’s first child; at age 54 in 1790, otherwise between ages 50 and 53, r_s_ = -0.392, p = 0.120; son’s first child: between ages 54 and 56, r_s_ = -0.393, p = 0.119). Average age for any grandchild’s birth followed a largely similar pattern: 60/61 years for maternal grandmothers, and 62 to 64 years for paternal grandmothers.

#### Number of grandchildren

**Fig 5:**
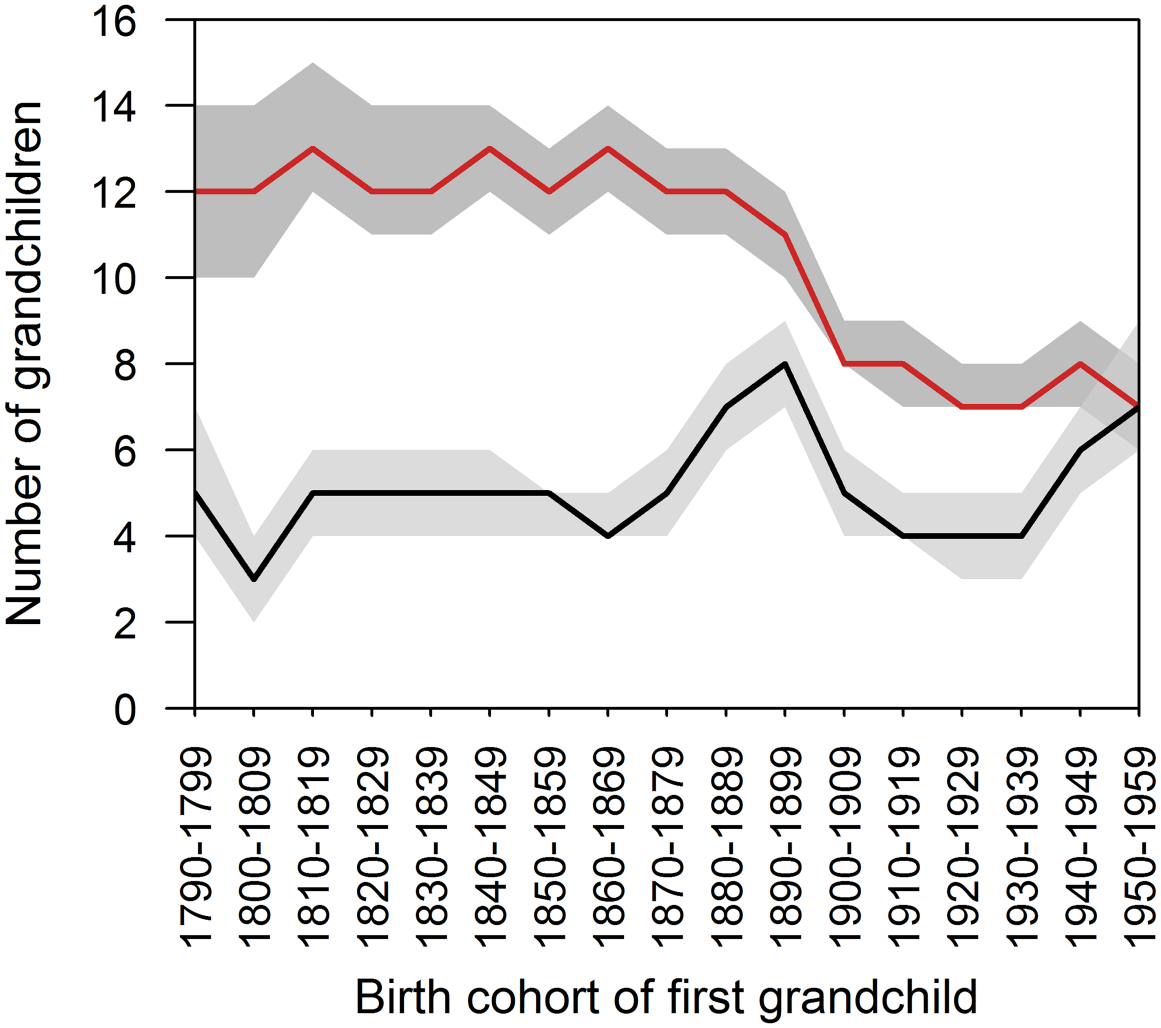
Number of grandchildren by birth cohort of first-born grandchild. Red line represents total number of grandchildren, black line the number of grandchildren born during the grandmother’s life. Grey areas indicate 95% confidence intervals.

We then determined whether potential grandmother investment and its costs changed through time due to changes in the number of grandchildren a grandmother had available to care for (and potentially competing over her investment). We found that, over the period of study, the total number of grandchildren born to a grandmother declined (r_s_ = -0.801, p<0.001). For those who became grandmothers for the first time from the 1790s to the 1870s, the average number of grandchildren was consistently high, fluctuating between 12 and 13 (Fig 5). The average number born during a grandmother’s life was similarly consistent, albeit much lower: between 3 and 5 for this same period (Fig 5). Numbers of total grandchildren stayed high for some years after the onset of the demographic transition—11 for the 1890s cohort. The average number of grandchildren born during the lifetime of a grandmother increased briefly to 8 in the 1890s, indicating grandmother longevity had increased as average age at birth/first birth did not change much over the study period, then decreased again to 4 or 5 for the early 20^th^ century; there was no significant change from the 1790s to 1950s in either direction (r_s_ = 0.198, p = 0.446). Birth rates appear to have declined going into the 20^th^ century, as indicated by lower total numbers of grandchildren—an average of 7 or 8 for all cohorts from 1900s onwards. The difference between the total number of grandchildren and the number born during a grandmother’s life decreased greatly (r_s_ = -0.854, p<0.001), with no difference by the 1950s; the average grandmother would then live long enough to see all grandchildren born.

#### Proximity to grandchildren

**Fig 6:**
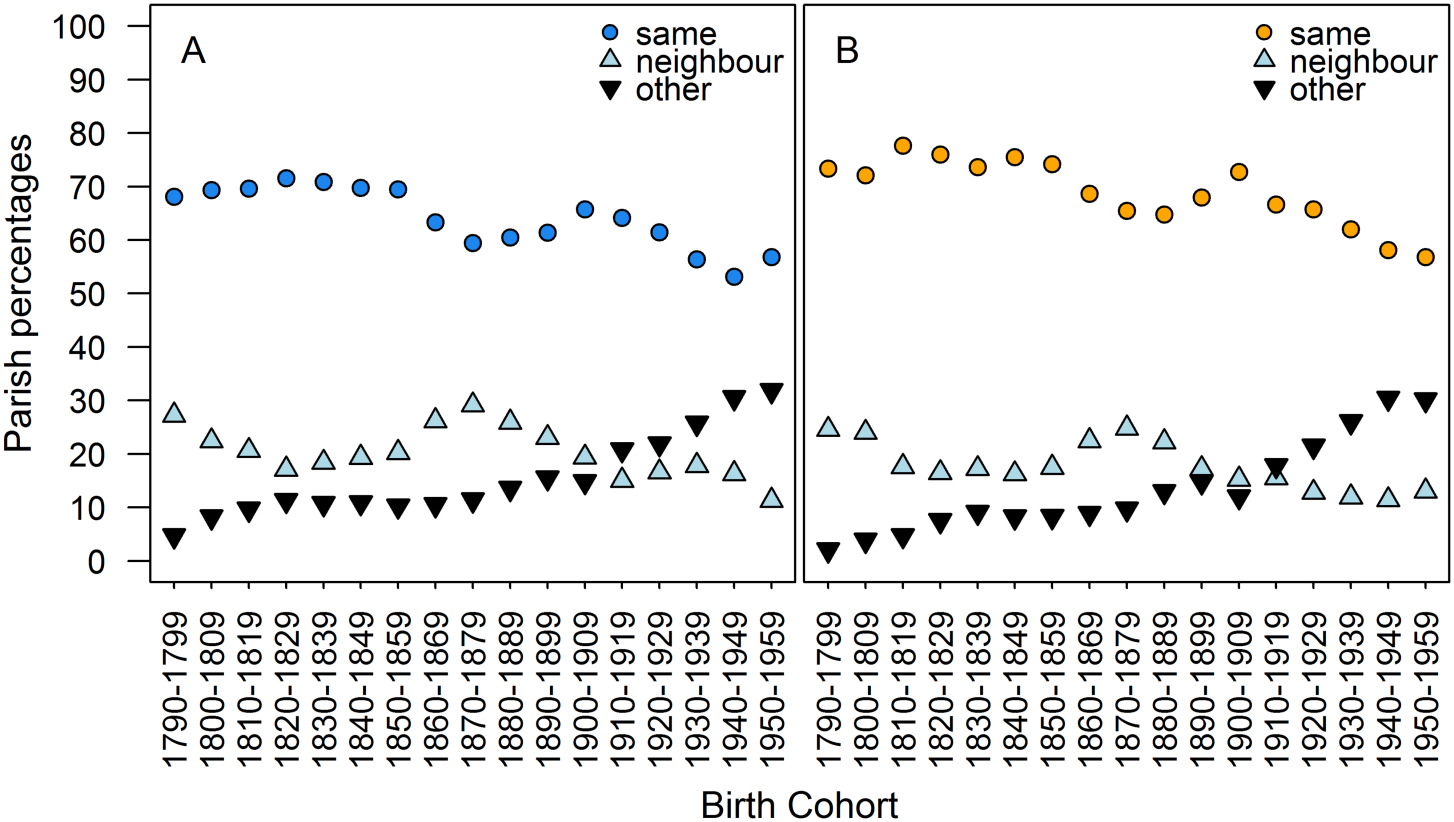
Proximity of grandmothers to grandchildren. a) Maternal grandmothers, b) paternal grandmothers. Circles indicate maternal (blue) and paternal (orange) grandmothers in the same parish, light blue triangles indicate grandmothers in neighbouring parishes, and black inverted triangles indicate grandmothers in different, non-neighbouring parishes.

Finally, we found evidence of changing residence patterns through time, which would have greatly affected the potential of grandmothers to directly interact with their grandchildren. Though historical data does not allow accurate quantification of the exact nature and amount of help grandmothers might have provided to their grandchildren, it is highly likely that those living far away from their grandchildren would have been able to provide less help than those within the same parish. We found that the percentage of grandmothers living in the same parish as grandchildren was greater for paternal grandmothers than maternal grandmothers, as would be expected in a patrilocal society (Wilcoxon signed rank test p<0.001). This percentage declined for both maternal grandmothers and paternal grandmothers over the study period (maternal grandmothers r_s_ = -0.792, p<0.001; paternal grandmothers r_s_ = -0.811, p<0.001; Fig 6), from 73.4% in the 1790s to 56.8% in the 1950s for paternal grandmothers, and 68.1% in the 1790s to 56.9% in the 1950s for maternal grandmothers. From the 1790s to the 1950s, the number living in neighbouring parishes decreased too (maternal grandmothers r_s_ = -0.564, p = 0.020; paternal grandmothers r_s_ = -0.750, p<0.001), from 27.2% to 11.2% (maternal grandmothers), and from 24.6% to 13.0% (paternal grandmothers).

## Discussion

Grandmothers are important allomothers in many societies, but the level of care they have provided both in historic and contemporary populations is limited by the shared time between the generations. However, despite the current challenges arising from ageing populations around the world, little work has investigated variation in the length of grandmotherhood through time, or its underlying drivers. We investigated changes in grandmotherhood over the demographic transition in Finland, finding the number of years that grandmothers and grandchild lived simultaneously drastically changed from the end of the 18^th^ century, well into the middle of the 20^th^ century. Our result provide historical context to the length of contemporary grandmother-grandchild relationships, which can be useful for a number of fields.

First, the changing length of grandmotherhood is of interest to our understanding of evolutionary factors promoting family living and post-reproductive life in the past. In our study population, grandmother presence has been shown to be particularly beneficial for grandchild survival for a period after weaning (ages 2–5 years) [12]. In light of this, our finding that, throughout the study period, grandmothers who were alive at the birth of a grandchild lived, on average, long enough (Fig 2) to cover the most critical period for helping grandchildren to survive is of interest. It also suggests the hypothesised fitness benefits of grandmotherhood [16] can still be met even if shared time is relatively low, and longer post-reproductive lifespan is potentially under selection in similar conditions. Although infant survival is no longer a key component in this intergenerational relationship in industrialised societies, where childhood mortality rates have generally been minimised by modern medical care, vaccination programs, and improved nutrition and hygiene, grandmother care has frequently been shown to influence grandchild outcomes [3,10–17]. Furthermore, grandmothering still contributes evolutionary fitness benefits (just not via childhood survival benefits), because grandmothering can reduce birth intervals and increase birth rates of adult daughters [13]. Regarding family living, though total grandchild numbers decreased, the number of grandchildren born to a grandmother when she was alive was about the same at the beginning and end of our study period (Fig 5), suggesting that any increase to investment to each grandchild would mostly come from increases to shared time, and not from a grandmother having fewer grandchildren to divide investment between.

Second, sociological and anthropological work, particularly that investigating changing family structures and relations, should take longitudinal demographics such as those presented here into consideration. Our results, stemming from a pre-modern society in which extended family living was common through to a post-industrialisation period with nuclear families [44,45], show that the demographic transition was associated with large changes in shared time, and could indicate how the length of intergenerational relationships might change in contemporary societies either undergoing or yet to undergo the demographic transition. However, though potential time to help may increase because of the improved survival and longevity of grandmothers and grandchildren [50], it can also be limited by fertility decisions that delay grandparenthood [29,30,51]. Furthermore, changes in family structure could affect how much of this potential helping time is realised. During the period in which shared time was low, grandmothers lived closer to their grandchildren than they did after shared time began to increase, but extensive motorisation only occurred towards the mid-20^th^ century and the rail network was mainly between the large population hubs [52]; long-distance travel was therefore limited for much of our study period, and only grandmothers living nearby were likely to have been able to provide help. This serves to highlight the necessity of investigating other contextual factors that can limit help, as shared time is only the maximum possible investment period, and alone cannot inform greatly on grandmother investment. Grandmother morbidity, for example, could reduce the amount of shared time in which a grandmother is physically or mentally capable of caring [51]. As industrialised societies have tended towards the nuclear family structure for much of the 20^th^ century [33], it may be the case that actual grandmother investment is no higher than in the past, and that investment will increase to match shared time as the prevalence of the nuclear family declines [33].

Third, grandmothering time is of increasing importance in the fields of population health and economics, though this depends heavily on the burden of grandmotherhood to grandmothers (i.e. how many grandchildren, and how much help is required). We have shown that the amount of shared time between grandmothers and their grandchildren has increased greatly, likely driven by decreasing childhood mortality. As the constraints on this shared time imposed by grandmother longevity alleviate as well, health benefits [14–17,21–23] and costs [19,21,22,24] of grandmothering may be exacerbated. People in contemporary industrialised societies are generally living longer and healthier lives than in the past, due to medical advances, hygiene practices, and social care, which not only leads to increased shared time, but also possibly to longer periods of healthy grandmothering [51]. It is concerning though that greater childcare demands could actually be costly in terms of grandmother health. This would consequently affect the economy, as there is a higher need for medical care, and grandmothers are out of the workforce. Populations are already ageing such that demographic structures are skewing towards older ages in developed countries [53], which has economic implications on national scales too [53], as there is difficulty finding viable financial solutions to take care of all elderly people; when childcare is detrimental to the health of grandmothers, a parent may have to leave work to care for them. Furthermore, labour force participation is already much lower for women than men in many countries, and this is in part a consequence of lack of affordable childcare [54]; either a mother remains home and cannot return to work, and is therefore out of the workforce, or one or both grandmothers take on childcare duties and are out of the workforce. Indeed, addressing the issue of childcare and retention of women in the workforce is currently one of the European Commission’s priorities [54]. Furthermore, later retirement is economically preferable at a population level to ensure the solvency of pensions plans [55], so understanding past and current trends in shared time is of economic and global importance.

The underlying factors determining the length of shared time between grandmothers and grandchildren in each population are existing mortality and fertility patterns. Here, the mortality constraints on shared time decreased over the study period (as expected after the demographic transition), as seen in two measures: the percentage of grandmothers alive at birth, and the percentage of grandchildren outliving their grandmothers, indicating greater survival of grandmothers in old age and greater survival through childhood, respectively. The timing of fertility also has a substantial effect on the length of shared time, especially in modern populations [28,29]. Age at birth can indicate fertility decisions in a society, and sets a soft limit on shared time: the later birth is, the less time there is left of a grandmother’s lifespan. Unlike contemporary Western populations, in which grandparenthood is being delayed due to fertility decisions [29,30,51], age at birth did not have a role in the change in shared time in Finland. It remained fairly consistent across the entire 170 year span for parents and grandmother, despite extreme social change in the form of declining birth rates [56] and the advent of birth control methods. This may be a downstream consequence of both late marriage [57] and of the rarity of pre-marital births for most of the study period. Historical birth timings are particularly important for future intergenerational relationships, as it can affect the onset of grandparenthood for the parental generation. For these demographic markers, there was a small, but fairly consistent, difference between maternal and paternal grandmothers, with paternal grandmothers less present for grandchildren. This is a likely reflection of population-level differences in the age of marriage of men and women; paternal grandmothers are older at the birth of their first grandchild, so will (on average) have a shorter shared time than their maternal counterparts.

We document here a transition to more common grandmother prevalence and a greater shared time between generations, showing that there can be large temporal differences within one country. Between-population variation in grandmother effects/investment could, in addition to cultural and societal differences, reflect geographical differences in the progress of the demographic transition and adult longevity modifying how common grandmother-grandchild interactions could have been. We therefore encourage other studies on longitudinal, multigenerational datasets (e.g. from parish records), particularly those in which grandmother effects have been shown, to investigate the role of grandmotherhood demography as a mediator of grandmothering outcomes.

## Supporting information

S1 Dataset Numbers of individuals by cohort for calculating proportions. (CSV)

S2 Dataset Individual-level information for cox models. (CSV)

S1 R Code Annotated R code used for all analyses. (R)

S1 File Explanations of variables in S1 and S2 Datasets used in S1 R Code. (DOCX)
