# Supplementary material for: Grandmotherhood across the demographic transition
Source: PLoS One. 2018 Jul 23;13(7):e0200963. doi: 10.1371/journal.pone.0200963 (PMC6056041; doi:10.1371/journal.pone.0200963)
Supplement: S1 File — (DOCX) [file pone.0200963.s004.docx]

This supplementary file is a codebook explaining each variable in files S1 Dataset and S2 Dataset. Please also refer to S1 R Code.

Analyses will be referred to in brackets (following the name of each variable) by their number in S1 R Code, with line numbers referring to the lines numbers in S1 R Code:

(1) Percentage grandmothers alive at birth (lines 42-62)

(2) Shared time (lines 63-180)

(3) Percentage grandchildren outliving grandmothers (lines 181-198)

(4) Grandmother age at grandchild birth (lines 199-327)

(5) Numbers of grandchildren (lines 328-383)

(6) Percentage living in same parish (lines 384-407)

**S1 Dataset** - these variables are population-level

**bcohort** (1)(3)(6) - this variable is used in all the analyses using S1 Data, and is simply each decade in the study period

**bcohort_mgm** (1) **-** total number of grandchildren in each birth decade with a maternal grandmother used in this analysis

**bcohort_alive_mgm** (1) **-** number of grandchildren in each birth decade with a maternal grandmother alive at their birth

**bcohort_pgm** (1) **-** total number of grandchildren in each birth decade with a paternal grandmother used in this analysis

**bcohort_alive_pgm** (1) **-** number of grandchildren in each birth decade with a paternal grandmother alive at their birth

**mgm_num_outlive** (3) - total number of grandchildren in each birth decade with a maternal grandmother used in this analysis

**mgm_death_before** (3) - number of grandchildren in each birth decade who died before their maternal grandmother

**pgm_num_outlive** (3) - total number of grandchildren with a paternal grandmother used in this analysis, by birth decade

**pgm_death_before** (3) - number of grandchildren in each birth decade who died before their paternal grandmother

**parish_mgm_num** (6) - total number of grandchildren in each birth decade with a maternal grandmother used in this analysis

**parish_mgm_same** (6) - number of grandchildren in each birth decade who lived in the same parish as their maternal grandmother

**parish_mgm_diff** (6) - number of grandchildren in each birth decade who lived in a different (and non-neighbouring) parish to their maternal grandmother

**parish_mgm_neigh** (6) - number of grandchildren in each birth decade who lived in a neighbouring parish to their maternal grandmother

**parish_pgm_num** (6) - total number of grandchildren in each birth decade with a paternal grandmother used in this analysis

**parish_pgm_same** (6) - number of grandchildren in each birth decade who lived in the same parish as their paternal grandmother

**parish_pgm_diff** (6) - number of grandchildren in each birth decade who lived in a different (and non-neighbouring) parish to their paternal grandmother

**parish_pgm_neigh** (6) - number of grandchildren in each birth decade who lived in a neighbouring parish to their paternal grandmother

**S2 Dataset** - these variables are individual level

**mgm_bcohort_share** (2) - the birth decade of each maternal grandchild used in this analysis

**mgm_shared_all** (2) - the shared time in years for each maternal grandchild. This was done by comparing the death year (or year last recorded) for the grandmother and grandchild. The earliest year was then subtracted from the birth year of the grandchild. If this was below 0, the shared time was set to 0 (see Methods)

**mgm_shared_2** (2) - the shared time in years for each grandchild whose maternal grandmother was alive at their birth. This was done by comparing the death year (or year last recorded) for the grandmother and grandchild. The earliest year was then subtracted from the birth year of the grandchild

**mgm_shared_event** (2) - an event variable for use in the *Surv* function for the cox model. 1 indicates the shared time was limited by death of either the maternal grandmother or grandchild, and 0 indicated the grandmother or grandchild had disappeared from the records before the death of either of them (i.e. censored)

**pgm_bcohort_share** (2) - the birth decade of each paternal grandchild used in this analysis

**pgm_shared_all** (2) - the shared time in years for each paternal grandchild. This was done by comparing the death year (or year last recorded) for the grandmother and grandchild. The earliest year was then subtracted from the birth year of the grandchild

**pgm_shared_2** (2) - the shared time in years for each grandchild whose paternal grandmother was alive at their birth. This was done by comparing the death year (or year last recorded) for the grandmother and grandchild. The earliest year was then subtracted from the birth year of the grandchild

**pgm_shared_event** (2) - an event variable for use in the *Surv* function for the cox model. 1 indicates the shared time was limited by death of either the paternal grandmother or grandchild, and 0 indicated the grandmother or grandchild had disappeared from the records before the death of either of them (i.e. censored)

**mgm_bcohort_aab** (4) - the birth decade of each maternal grandchild used in this analysis

**mgm_aab** (4) - the maternal grandmother’s age at the birth of the focal grandchild, calculated by subtracting the grandchild’s birth year by the grandmother’s birth year

**pgm_bcohort_aab** (4) - the birth decade of each paternal grandchild used in this analysis

**pgm_aab** (4) - the paternal grandmother’s age at the birth of the focal grandchild, calculated by subtracting the grandchild’s birth year by the grandmother’s birth year

**mgm_bcohort_aab_fi** (4) - the birth decade of each first-born maternal grandchild

**mgm_aab_fi** (4) - the maternal grandmother’s age at the birth of the focal first-born grandchild, calculated by subtracting the grandchild’s birth year by the grandmother’s birth year

**pgm_bcohort_aab_fi** (4) - the birth decade of each first-born paternal grandchild

**pgm_aab_fi** (4) - the paternal grandmother’s age at the birth of the focal first-born grandchild, calculated by subtracting the grandchild’s birth year by the grandmother’s birth year

**gm_bcohort** (5) - the birth decade in which a grandmother’s first grandchild was born. This was done by first listing all a grandmother’s grandchildren, then selecting the earliest year of birth

**gm_total** (5) - the total number of grandchildren of that grandmother. This was done by first listing all of a grandmother’s grandchildren and then by summing the number of rows of grandchildren. No grandchildren were excluded

**gm_alive** (5) - the number of grandchildren born to a grandmother whilst she was known to be alive. This was calculated by summing the number of grandchildren with a year of birth before the grandmother’s year of death/year of disappearance (if the latter, her **gm_event** value was set as 0; see below)

**gm_event** (5) - an event variable for use in the *Surv* function for the cox model. 1 indicates the grandmother had a known year of death, and 0 indicated they were censored
